# Identification of Key Aroma-Active Compounds in Commercial Coffee Using GC-O/AEDA and OAV Analysis

**DOI:** 10.3390/foods14183192

**Published:** 2025-09-13

**Authors:** Xiaomei Chen, Panpan Wu, Shuwei Wang, Jie Sun, Haitao Chen

**Affiliations:** Beijing Key Laboratory of Flavor Chemistry, Beijing Technology and Business University, Beijing 100048, China; 18732002674@163.com (X.C.); 18617240636@163.com (P.W.); wsw3325@163.com (S.W.)

**Keywords:** volatile compounds, GC-O, AEDA, odor activity value, aroma recombination

## Abstract

In this study, we systematically characterize the volatile and non-volatile flavor profiles of coffee beans. Sensory evaluation demonstrated unique aromatic profiles for each coffee, with Colombia excelling in chocolate and nutty notes, while Bench Maji exhibited pronounced fruity characteristics. Yirgacheffe had a prominent roasted aroma, and Baoshan stood out for its stronger woody and caramel notes. The analysis employed solvent-assisted flavor evaporation (SAFE), gas chromatography–mass spectrometry/olfactometry (GC-MS/O), and high-performance liquid chromatography (HPLC). A total of 85 aroma compounds were identified, with furans, ketones, and pyrazines being the predominant contributors to roasted, nutty, and caramel aromas. Key aroma-active compounds, including furfural, guaiacol, and furaneol, exhibited the highest flavor dilution (FD) factors (up to 2187) and odor activity values, highlighting their pivotal roles in coffee aroma, with 4-vinyl-2-methoxyphenol (OAV = 761 in GL) and furaneol (OAV = 250 in BS) being particularly influential. Recombination and omission experiments validated the significance of these compounds. Non-volatile analysis revealed distinct differences in organic acids and chlorogenic acid content, with Colombia samples showing higher quinic acid levels, likely due to roasting-induced degradation. These findings provide a theoretical basis for understanding coffee flavor diversity and offer insights for quality assessment and origin identification.

## 1. Introduction

Coffee is one of the most widely consumed beverages globally and the second most traded commercial commodity [1]. The aroma of coffee, a key determinant of its quality and consumer preference, is generated by volatile compounds formed during roasting [2,3]. To date, over 1000 volatile compounds have been identified in coffee [4], with furans, pyrazines, ketones, and phenols being the most significant contributors to its distinctive aroma. These compounds are influenced by multiple factors, including coffee species and cultivars, growing conditions, post-harvest processing, blending, roasting techniques, and storage [5]. In addition to volatile compounds, the sensory profile of coffee is also shaped by non-volatile constituents [6]. Among these, caffeine is one of the most studied bioactive substances and is also recognized as a bitter flavor enhancer in coffee [7]. Acid is regarded as a key factor in the sensory experience of coffee. The acids in coffee are generally divided into organic acids (OAs) and chlorogenic acids (CGAs) [8]. Each organic acid contributes uniquely to coffee’s taste, aroma, and overall sensory profile [9]. From a compositional perspective, both volatile and non-volatile compounds significantly influence coffee’s flavor perception, consumer acceptance, and enjoyment [10].

Coffee-growing regions are primarily distributed in Africa, the Americas, and Asia, with coffee beans from different countries and regions exhibiting distinct flavor characteristics [11]. As consumer interest in specialty coffee grows, understanding the chemical basis of these sensory profiles becomes increasingly important for quality assessment and product development. The two most economically significant coffee species are Arabica and Robusta [12]. Each has unique sensory attributes; Arabica coffee is characterized by high acidity and rich fruity aromas, while Robusta has higher caffeine levels and greater bitterness [13]. Arabica’s more complex flavor profile also contributes to its higher consumption compared to Robusta [14]. Previous studies have characterized volatile compounds in roasted Arabica or Robusta coffees using targeted approaches. Pua et al. and Shi et al. characterized and identified the volatile components in roasted Arabica coffee beans from different countries through GC-MS and GC-O techniques [11,15]. Dong et al. conducted a systematic analysis of the chemical composition of seven Robusta coffee varieties in Hainan Province [16]. Additionally, Freitas and Mosca, n.d., explored the aroma components of Arabica and Robusta coffee from different geographical sources [17]. However, studies on the key aroma compounds in coffee beans and their associations with sensory attributes remain limited.

In recent years, extraction methods for volatile compounds have predominantly concentrated on solid-phase microextraction (SPME) and solvent-assisted flavor evaporation (SAFE) [18,19]. SAFE generally enables the extraction of a greater diversity of volatile compounds compared to headspace techniques [20]. However, its application in coffee flavor research remains relatively limited. Gas chromatography–mass spectrometry (GC-MS) is the most widely used technique for detecting volatile compounds in foods [21]. Gas chromatography–olfactometry (GC-O) has proven effective for the extraction and identification of key aroma-active compounds. The combination of aroma extract dilution analysis (AEDA) with odor activity value (OAV) analysis provides guidance for determining critical flavor substances [22]. Furthermore, recombination and omission experiments have been employed to validate the contribution and influence of individual compounds to overall aroma profiles [23]. However, comprehensive studies integrating these methodologies to compare coffees from diverse geographical origins remain limited. Czerny et al. first introduced the recombination–omission model in coffee research, but only 28 key aroma compounds were quantitatively analyzed; potential contributions from unexamined compounds, such as specific pyrazines, require further investigation [24].

This study systematically characterized the volatile and non-volatile composition of Arabica beans from four different areas (Colombia, Bench Maji, Yirgacheffe, and Baoshan) using an integrated analytical approach. Volatile aroma compounds were extracted by solvent-assisted flavor evaporation (SAFE) and analyzed through gas chromatography–mass spectrometry/olfactometry (GC-MS/O), with key aroma-active compounds identified via aroma extract dilution analysis (AEDA) and odor activity value (OAV) calculations, followed by sensory validation through recombination and omission experiments, aiming to elucidate the differential patterns of key aroma-active compounds among coffee samples with distinct sensory attributes. The organic acids in the non-volatile components of coffee were determined by liquid chromatography–mass spectrometry (LC-MS), while caffeine and chlorogenic acid were determined by high performance liquid chromatography (HPLC) to illustrate the influence of non-volatile components on the aroma of coffees with different sensory characteristics. This study systematically compared flavor compositions among coffee beans with distinct sensory attributes, enabling the precise identification of key aroma compounds responsible for their sensory differentiation. Our findings elucidate the functional roles of these compounds in shaping characteristic flavor profiles, establishing a scientific foundation for optimizing coffee processing techniques and enhancing product quality.

## 2. Materials and Methods

### 2.1. Coffee Samples

Colombia coffee bean samples were purchased from Nestlé Products (Shanghai) Services Co., Ltd. (Shanghai, China). Bench Maji and Yirgacheffe coffee bean samples were purchased from Luckin Coffee Technology (Hainan) Co., Ltd. (Haikou City, China). Baoshan coffee bean samples were purchased from Baoshan Zhongka Food Co., Ltd. (Baoshan City, China). They are all 100% Arabica and were processed by the washed method. The roasting degree of all coffee beans was medium, with similar parameters maintained throughout the roasting process. Colombia coffee beans had a roasting temperature of 205 °C and a roasting time of approximately 8 min; Bench Maji coffee beans had a roasting temperature of 195 °C, with a roasting time of 11 min; and Yirgacheffe and Baoshan coffee beans were roasted at 200 °C for 12 min and at 202 °C for 10 min, respectively. The coffee beans were ground into powder with Kin Grinder K6 and sifted through a 20-mesh sieve for analyzing. The samples of ground coffee powder are shown in Figure S1.

### 2.2. Chemicals

Dichloromethane and sodium sulfate were purchased from Mreda (Beijing, China). 2-acetylpyridine, levulinic acid, (S,S)-2,3-butanediol, acetylpyrazine, 2-methyltetrahydro-3-furanone,2,3-butanediol,(R)-3-hydroxy-4,4 dimethyldihydrofuran-2(3H)-one,2-(hydroxymethyl)thiophene,3,4-dimethoxystyrene, and tetrahydrofurfuryl alcohol were purchased from Accela ChemBio Co., Ltd. P-xylene, pyrrole, furfuryl acetate, 4-hydroxypyridine, and dibutyl phthalate were purchased from Shanghai Acmec Biochemical Technology Co., Ltd. Butyric acid, 2,6-dimethylpyrazine, 2-methyl-2-cyclopenten-1-one, 4-ethylphenol, dihydro-2-methyl-3(2H)-thiophenone, 2-ethyl-6-methylpyrazine, 1-furfurylpyrrole, cis-2-penten-1-ol, 1-ethyl-1h-pyrrole-2-carbaldehyde, 6,7-dihydro-5-methyl-5(H)-cyclopentapyrazine, pyrazine, (2E,4E)-octa-2,4-dienal, 3-mercapto-3-methyl-1-butanol, 4,5-dimethylthiazole, 2,3-hexanedione, 2-vinylpyrazine, 3,4-hexanedione, 2-methylhexanoic acid, 3-hexanone, acetoxy-2-propanone, 2-pyrrolidinone, furan-2-ylmethyl propionate, n-hexyl formate, ethyl-beta,beta-dimethyl acrylate, 4-methylvaleric acid, 2-methyl-2-butanol, propionic acid, (+)-pulegone, 2-acetyl-1-methylpyrrole, and furfuryl alcohol were purchased from Adamas (Shanghai, China). Ethylbenzene standard, myrcene, 2,3,5-trimethylpyrazine, 2,2-dimethyl-3-hexanone, and 2-methyl-3-pentanone were purchased from Aladdin (Shanghai, China). 2-ethylpyridine and guaiacol were purchased from Innochem (Beijing, China). 2-(furan-2-ylmethyl) furan, 3-ethyl-2,5-dimethylpyrazine, 2-methyl-1-butanol acetate, and 3-methyl-3-buten-1-ol were purchased from Macklin (Shanghai, China). Pyridine, 2-methyl-3-buten-2-ol, 2-(methoxymethyl)furan, hexanoic acid, 2-((methylthio)methyl)furan, 2-ethyl-5-methylfuran, 2,4,5-trimethyloxazole, 3,5-dihydroxy-6-methyl-2H-pyran-4(3H)-one, (+)-Limonene, and N-methyl pyrrole were purchased from Energy Chemical (Shanghai) Co., Ltd. (Shanghai, China).

### 2.3. Color Measurements

The color of the roasted coffee grounds was quantified by measuring its CIELAB values using a spectrophotometer (CS-580, Hangzhou CHNSpec Technology Co., Ltd., Hangzhou, China). Results are expressed on the C.I.E. L*a*b* scale L* (lightness), a* (red–green value), and b* (yellow–blue value). Each treatment had five replicates.

### 2.4. Determination of Volatile Substances

#### 2.4.1. Isolation of Volatiles by Solvent-Assisted Flavor Evaporation (SAFE)

Coffee powder (50 g) was extracted with 200 mL dichloromethane, and then shaken at 300 rpm for 30 min. After filtration, the mixture was spiked with internal standard 3-heptanone (20 μL, 81.8 mg/mL). The volatile compounds of coffee were isolated using SAFE with a high vacuum. Liquid nitrogen was continuously added to cold traps and thermos flasks. The temperature of the water bath was maintained at 50 °C. The extract was dried by anhydrous Na_2_SO_4_. After filtration, it was distilled to about 2 mL through a Vigreux column (50 × 1 cm; Beijing Jingxing Glassware Co., Ltd., Beijing, China). The final distillate was concentrated to 1 mL under a gentle flow of nitrogen for subsequent analysis.

#### 2.4.2. GC-MS Analysis

GC-MS analysis was undertaken using a Thermo Fisher Trace 1310 GC system configured with a single quadrupole mass spectrometer (both Thermo Fisher Scientific, Waltham, MA, USA). Separation was achieved on a TG-Wax capillary column (30 m × 0.25 mm i.d., 0.25 μm film thickness; Thermo Fisher Scientific) with helium carrier gas at a constant flow rate of 1.0 mL/min. The column temperature was programmed as follows: from 400 °C (2 min) to 1300 °C (1.55 °C per min), from 1300 °C to 2200 °C (44 °C per min), and then 2200 °C for 5 min; the total run time was 89.5 min. The analysis was performed with a 1 μL injection volume (split ratio 1:20). MS parameters: 70 eV EI ionization, 230 °C ion source, 230 °C transfer line. Mass range: *m*/*z* 35–300 with 4 min solvent delays.

### 2.5. Determination of Non-Volatile Substances

#### 2.5.1. Organic Acid Analysis

The analysis process is as follows: Weigh 0.5 g of coffee powder, add 30 mL of 50% ethanol–water solution (*v*/*v*), and perform ultrasonic extraction for 40 min. After centrifugation, collect the supernatant and dilute it to 50 mL with 50% ethanol–water solution. Filter the solution through a membrane, then dilute the filtrate with 0.1% formic acid in methanol–water (1:9, *v*/*v*) for subsequent analysis. The quantitative analysis of quinic acid, succinic acid, and lactic acid was performed using a Waters I-Class UPLC system coupled with a Waters XEVO-TQS micro mass spectrometer. The chromatographic separation was achieved on an ACQUITY UPLC BEH C18 column (2.1 × 100 mm, 1.7 μm) maintained at 35 °C, with the sample compartment temperature set at 20 °C. A 5 μL injection volume was used, and the mobile phase consisted of (A) 0.1% formic acid in water and (B) 0.1% formic acid in methanol, delivered in gradient mode. For the analysis of citric acid, malic acid, and tartaric acid, the same UPLC-MS system was employed but with an ACQUITY UPLC Peptide BEH C18 column (2.1 × 150 mm, 1.7 μm) under identical temperature conditions. The injection volume was 2 μL, and the mobile phase was (A) 0.05% ammonia in water and (B) methanol.

#### 2.5.2. Caffeine and Chlorogenic Acids Analysis

Caffeine determination was conducted using a Shimadzu LC-20AD HPLC system with PDA detection. Exactly 0.5 g of sample was placed in a 250 mL conical flask, extracted with 80 mL of ultrapure water at 80 °C for 30 min, and then cooled. After adding 2 g of magnesium oxide and shaking, the mixture was reheated for 20 min before final cooling and filtration through a 0.22 μm membrane into a 2 mL Agilent vial. Separation was performed on a GIST-C18 column (4.6 × 250 mm, 5 μm) at 35 °C with 1 mL/min flow rate and a 10 μL injection volume. The mobile phase consisted of (A) 0.1% phosphoric acid in water and (B) methanol, delivered in gradient mode.

The quantification of chlorogenic acid was performed using a Thermo Fisher Scientific HPLC system equipped with a PDA detector. Precisely 0.5 g of the sample was weighed into a 25 mL volumetric flask, extracted with 20 mL of 70% methanol solution via ultrasonication for 30 min, and then brought to volume with the same solvent. After thorough mixing and centrifugation, the supernatant was filtered through a 0.22 μm membrane into a 2 mL Agilent vial for analysis. Chromatographic separation was achieved on an InertSustain-C18 column (4.6 × 250 mm, 5 μm), and the other conditions were the same as those for caffeine.

Quantification of all non-volatile compounds was achieved using external standard calibration curves. The details of all calibration curves are compiled in Supplementary Table S1.

### 2.6. Gas Chromatography–Olfactometry (GC-O) Analysis and Aroma Extraction Dilution Analysis (AEDA)

The GC-O-MS system consisted of a Trace GC-MS equipped with an ODP3 olfactory detector (Gerstel, Mülheim an der Ruhr, Germany). Chromatographic separation was performed on a TG-WAX column under identical conditions to those used in GC-MS analysis. The coffee volatile extract was serially diluted three-fold with dichloromethane (1:3, 1:9, 1:27, 1:81, …, 1:2187) and analyzed by GC-O under standard conditions. Trained assessors with demonstrated competency in odor characterization evaluated each dilution level, with triplicate measurements performed for all samples. The flavor dilution (FD) factor was defined as the highest dilution factor at which each aroma compound could be reliably detected by the sensory panel.

### 2.7. Identification and Quantitation of Volatile Compounds

The volatile compounds in coffee were identified by comparing their mass spectra (MS) in the NIST 17 mass spectral database, and then the RI was calculated for each volatile compound using the retention times of a homologous series of C_6_–C_28_ n-alkanes. After that, the identification accuracy was further verified using standard compounds (Std).

On the basis of AEDA, the quantitation of the aroma compounds with high FD factors (FD ≥ 9) was performed by constructing standard curves. Calibration standards were prepared by serial dilution of the stock solution in dichloromethane spiked with 3-Heptanone internal standard. Mass spectrometry was performed in the single ion monitoring (SIM) mode.

### 2.8. Calculation of Odor Activity Values (OAVs)

The odor activity value (OAV), calculated as the ratio of compound concentration to its odor threshold, quantifies the relative contribution of individual aroma constituents to the holistic sensory characteristics.

### 2.9. Recombination and Omission Experiments

The coffee was exhaustively extracted with dichloromethane through multiple extraction–filtration cycles until complete odor removal was sensorially confirmed, thereby obtaining an odorless matrix. Aroma recombination was performed by incorporating the key odor-active compounds (OAV > 1) into the odorless coffee matrix. Comparative sensory analysis between the recombinant model and original coffee was conducted by trained panelists using established descriptive evaluation procedures.

Omission testing was performed by preparing a series of deficient models, each lacking one target odorant from the full recombination matrix, to assess its specific sensory impact through comparative profiling, followed by triangle tests to assess perceptible differences.

### 2.10. Aroma Profile Evaluation

The sensory evaluation panel consisted of 18 judges aged 20–28 years with training in quantitative descriptive analysis (QDA), recruited from the Beijing Key Laboratory of Flavor Chemistry at Beijing Technology and Business University. All trained panelists were experienced in food sensory evaluations.

To characterize the aromatic profile of coffee, samples were placed in a covered odorless PET vial for sensory evaluation. A panel of trained experts conducted collaborative discussions to define the key odor descriptors, ultimately identifying seven dominant aromatic attributes: roasted, fruity, caramel, smoky, woody, chocolate, nutty, and floral. Each attribute’s perceived intensity was quantitatively assessed using a 10-point intensity scale (0 = not perceivable; 10 = strongly perceivable) during structured sensory sessions.

### 2.11. Statistical Analysis

Experimental data were collected and organized using Excel (Microsoft Office 2016, Redmond, WA, USA). The results of the experiments were expressed as the mean of three experiments ± standard deviation and analyzed by one-way analysis of variance using IBM SPSS version 27 (SPSS Inc., Chicago, IL, USA). Aroma profiles on the radar chart and the bar chart were plotted using Origin version 2024b (Origin Lab Corporation, Northampton, MA, USA).

## 3. Results

### 3.1. Color Value of Four Kinds of Coffee

To ensure that variations in roasting degree did not influence the analysis of aromatic compounds in coffee, colorimetric measurements were performed to standardize and verify the roast level. Based on CIELAB color space analysis of the four coffee samples, and with reference to the established literature [25,26,27], all samples were confirmed to exhibit colorimetric properties consistent with a medium roast. The L* (lightness) values ranged from 25.56 to 30.98, a* (red–green axis) values varied between 9.13 and 12.14, and b* (yellow–blue axis) values fell within 10.6 to 16.09 (Table 1). The low standard deviations (ranging from ±0.05 to ±0.6) observed across replicate measurements indicate high reproducibility and uniform roasting within each sample. These results confirm that the roasting degree was consistently maintained across all samples.

### 3.2. Sensory Analysis of Four Kinds of Coffee

**Figure 1:**
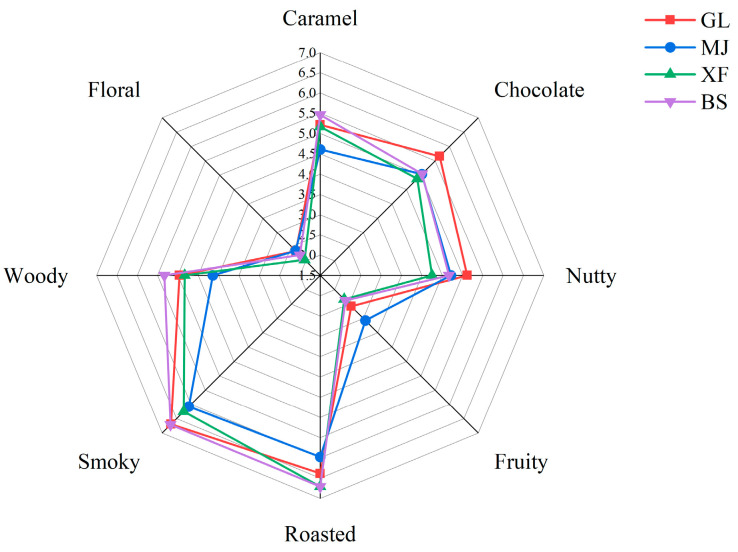
Aroma profile of four kinds of coffee.

The influence of growing conditions such as sunlight exposure and temperature resulted in distinct aromatic profiles among coffee samples from different origins, as revealed by quantitative descriptive analysis (QDA) evaluating eight sensory attributes (caramel, nutty, chocolate, floral, fruity, smoky, woody, and roasted). While the overall flavor profiles showed similarities, key differences emerged, as in Figure 1: GL exhibited superior chocolate and nutty characteristics compared to other samples, while BS displayed more pronounced woody and smoky notes. XF and BS demonstrated stronger roasted aromas but lower fruity intensity relative to the other two coffees. Notably, MJ outperformed all samples in fruity and floral aroma attributes while showing comparable or weaker performance in other flavor categories.

### 3.3. Volatiles in Four Kinds of Coffee

**Figure 2:**
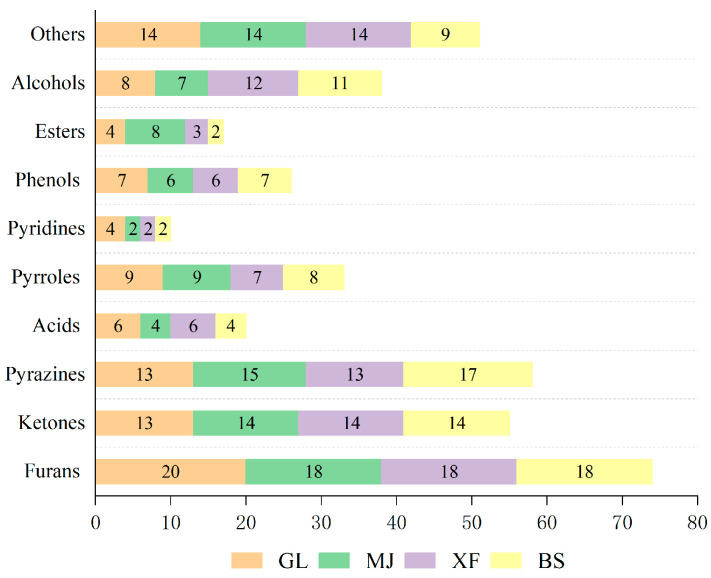
Comparison of the volatile compound types of coffee.

Volatile compounds from four coffee samples were extracted using solvent-assisted flavor evaporation (SAFE) and subsequently analyzed. A total of 132 volatile compounds were identified (the corresponding chromatogram is provided in Figure S2), with 67 constituents being common across all four samples. The GL and MJ samples exhibited greater volatile complexity, containing 99 and 97 compounds, respectively, compared to XF (95 compounds) and BS (92 compounds). The detected volatiles were classified into 10 chemical groups: 22 furans, 16 ketones, 19 pyrazines, 8 acids, 9 pyrroles, 4 pyridines, 7 phenols, 11 esters, 13 alcohols, and 22 others. A comparison of the volatile compound types of the coffees is shown in Figure 2. From the perspective of aroma compound composition, furans, ketones, and pyrazines were found to be the most abundant chemical classes among the volatile compounds identified in all four coffee samples. Overall, acids, pyridines, and ester compounds were low in the four kinds of coffee.

Furans and pyrazines are primarily generated through Maillard reactions and thermal degradation during roasting [28]. Among the furans, furfuryl alcohol and furfuryl acetate were found in all four coffee samples. They contribute to the bread and fruity aromas, respectively. Pyrazines usually have a relatively low odor threshold and are thus important aroma substances in coffee. Pyrazine compounds can bring nutty and earthy aromas to coffee products [29], among which methylpyrazine is the main component [25]. 2-methylpyrazine, 2,5-dimethylpyrazine, and 2,6-dimethylpyrazine are related to the cocoa, nut, and roasting aromas in coffee. Ketone compounds include hydroxyacetone, 1-hydroxy-2-butanone, and 2, 3-pentanedione. The presence of 2,3-pentanedione contributes to buttery and creamy aromas [21]. Pyridine and pyrrole compounds are the products of the thermal degradation of trigonubarine [11]. The main pyrrole compounds identified in all coffee samples included N-methyl-2-pyrrole formaldehyde, 2-acetyl-1-methylpyrrole, 2-acetylpyrrole, and 2-pyrrole formaldehyde. The main pyridine compounds were pyridine and 2-acetylpyridine. Among acid compounds, acetic acid, and isovaleric acid had a significant impact on the acidity in coffee. The presence of appropriate concentrations of acids contributes significantly to the desirable flavor profile of coffee. Phenolic compounds originate from the thermal degradation of chlorogenic acid [30], mainly including guaiacol, 4-ethyl guaiacol, and 4-vinyl guaiacol, which provide the smoky and woody aromas of coffee. In addition, there is maltol, which provides the caramel aroma. These are very important for the flavor of coffee. Among the alcohols, linalool and phenylethanol were present in four samples. They provided woody, citrus, and floral aromas, which contributed to enhancing the overall flavor of the coffee.

### 3.4. Non-Volatiles in Four Kinds of Coffee

**Figure 3:**
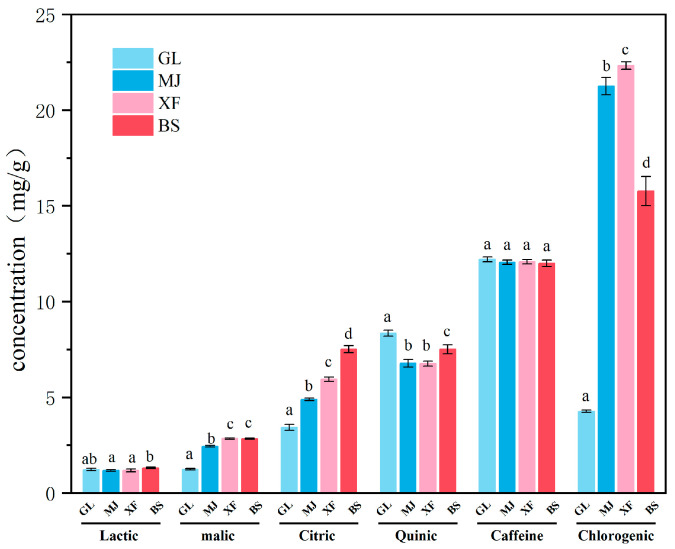
The content of organic acids, caffeine, and chlorogenic acid in coffee. Different letters indicate differences among samples (*p* ≤ 0.05) by Tukey’s test.

The LC-MS profiles of all coffee samples are shown in Figure S3. Quantitative analysis was performed on six common organic acids, caffeine, and chlorogenic acid in the four coffee samples, with results presented in Table 2 and visualized using clustered column charts in Figure 3. The total amounts of organic acids were GL 1.42%, MJ 1.53%, XF 1.67%, and BS 1.92%, respectively. Succinic acid and D-tartaric acid were not detected in all coffee (ND). Lactic acid is the source of the aromas of fruits, wines, and fermentation [31], and it did not vary significantly among different coffees. The content of malic acid was the lowest in the GL, and the contents were similar in the other three coffees. Citric acid showed significant differences in all four coffee samples. Rune et al. also found significant differences in citric acid among different production areas [8]. The content of quinic acid was the highest in the GL, while it was relatively lower in the other three coffees. Quinic acid is one of the most bitter substances in coffee beans [32].

Chlorogenic acid can produce bitterness, sourness, and astringency during coffee brewing [31]. The chlorogenic acid content in the four coffee samples shows significant differences. The content in XF was the highest (2.23 ± 0.02%), followed by MJ (2.13 ± 0.05%), while that in GL was significantly lower than that in the other three coffees (0.43 ± 0.01%). This difference may be related to environmental factors such as altitude and light conditions. Chlorogenic acid degrades into quinic acid during the baking process [33]. It is speculated that the reason for the low content of chlorogenic acid and the high content of quinic acid in GL might be the degradation of chlorogenic acid. There was no significant difference in caffeine content among the four coffee samples, ranging from 1.20% to 1.22%.

### 3.5. Aroma-Active Compounds in Coffee

**Figure 4:**
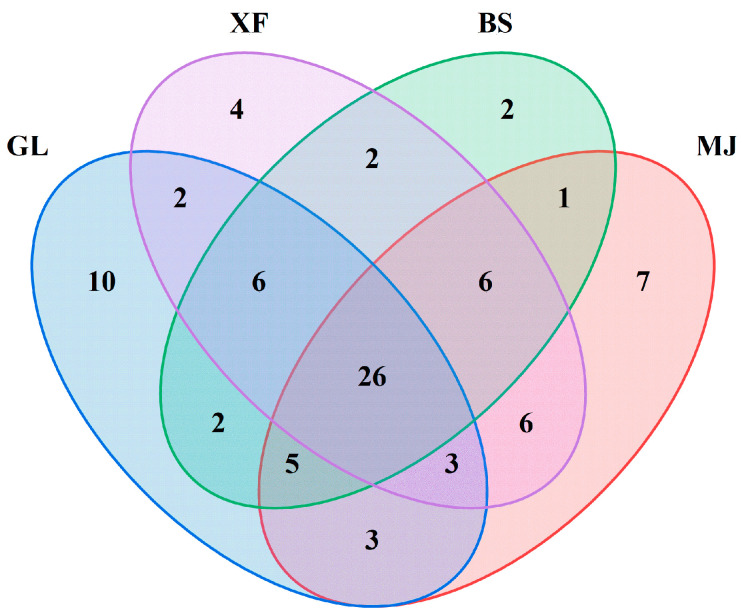
Venn diagram of aroma-active compounds in four coffee samples.

In order to identify the compounds that contribute to the overall aroma of coffee, characterization was carried out using GC-O combined with AEDA. The results are shown in Table 3. A total of 85 aroma compounds were detected, and their FD values ranged from 1 to 2187. The types of aroma compounds in different coffee samples vary greatly, resulting in different flavors. As shown in Figure 4, 57, 57, 55, and 50 flavor compounds were, respectively, found in the four coffee samples. There were 26 aroma substances existing simultaneously in the four coffees. Among them were 2-ethyl-5-methylpyrazine (FD = 81), furfural (FD = 2187), 2-acetylfuran (FD = 729), furfural acetate (FD = 729), 5-methylfurfural (FD = 243), γ-butyrolactone (FD = 243), isovaleric acid (FD = 243), 1-(2-furanomethyl)-1H-pyrrole (FD = 729), guaiacol (FD = 2187), 4-ethyl-2-methoxyphenol (FD = 729), furanone (FD = 729), and 4-vinyl-2-methoxyphenol (FD = 81); these substances have relatively high FD values, providing nutty, woody, caramel, and smoky flavors. They are therefore important contributors to the aroma of coffee.

The Venn diagram in Figure 4 illustrates the distribution of unique aroma-active compounds among the four coffee samples. Sample GL contained 10 distinctive aroma compounds, including three with particularly high FD factors: furfuryl methyl sulfide (FD = 2187), tetrahydrofurfuryl alcohol (FD = 243), and 2-(furan-2-ylmethyl)furan (FD = 81). MJ exhibited seven unique compounds, while XF possessed four unique compounds, notably 2-ethyl-3,5-dimethylpyrazine (FD = 27), with a relatively high FD value. BS showed the fewest unique compounds (2), among which 2,3-butanediol (FD = 243) demonstrated significant aroma potency. These sample-specific compounds, particularly those with elevated FD values, were found to contribute substantially to the distinctive aromatic profiles of each coffee.

### 3.6. Quantitation of the Aroma-Active Compounds and OAVs

**Figure 5:**
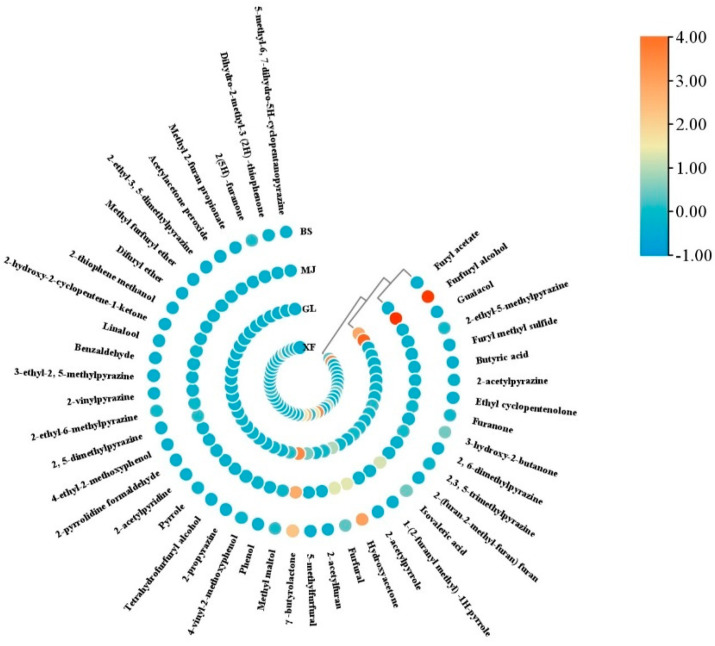
Heatmap of aroma compounds with FD ≥ 9 in four coffee samples.

The FD factor and OAV are usually used to measure the role of each compound in the overall aroma of food [34]. Based on the results of AEDA, 46 key aroma compounds with FD ≥ 9 in the four samples were accurately quantified. The R^2^ values of all standard curves were greater than 0.99, indicating a good fit of the linear equation (Table 4). In order to compare the differences among various coffee aroma compounds more clearly, a clustering heatmap was used to visualize the contents of these aroma compounds, as shown in Figure 5. The aroma compounds that contribute the most among the four kinds of coffee are furfuryl acetate, furfuryl alcohol, isovaleric acid, hydroxyacetone, furfural, 5-methylfurfural, and γ-butyrolactone, respectively. This is also the most crucial reason for the aroma differences among the four kinds of coffee. Furyl acetate has a sweet banana-like flavor, bringing a fruity aroma to GL. Furfuryl alcohol provides coffee with the aroma of bread. The content of furfuryl alcohol in XF is slightly lower than that in the other three kinds of coffee. The concentration difference in isovaleric acid in different coffees can significantly affect the flavor performance. At low concentrations, it brings a fermenting sensation and fruity aroma to the coffee, while at excessive concentrations, it brings an unpleasant flavor to the coffee. The difference in isovaleric acid content between MJ and GL leads to their distinct flavors. Hydroxyacetone brings a caramel aroma to coffee. The content of hydroxyacetone in BS and XF is higher than that in GL and MJ. Furfural provides coffee with a mixed aroma of almonds and toasted bread. It is an important volatile compound produced during the coffee roasting process. The contents of MJ and XF are significantly higher than those of BS and GL. γ-butyrolactone has the aroma of caramel and roasted nuts, with the highest content in GL, endowing it with a rich, nutty aroma.

However, the content of aroma components alone cannot be used as a basis for determining the aroma characteristics of coffee. Usually, it is the aroma components with a higher OAV that give coffee its aroma characteristics [36]. Compounds with larger OAVs are considered to contribute more to the overall odor characteristics of complex odor mixtures [34]. In order to identify the compounds that contribute significantly to the coffee odor, the OAV of these compounds with high FD was calculated. A total of 25 compounds with OAV > 1 were screened out, and 20, 18, 16, and 17 key volatile compounds were found in GL, MJ, XF, and BS, respectively. Among them, the OAVs of furfuryl alcohol, γ-butyrolactone, hydroxyacetone, isovaleric acid, furfural, 4-vinyl-2-methoxyphenol, furanone, guaiacol, and maltol in the four kinds of coffee were all greater than 1. According to GC-O analysis, the odor characteristics of these compounds include bread, nut, caramel, and baking, and smoky, woody, and fruity aromas. In GL, compared with the other three samples, the OAV of 4-vinyl-2-methoxyphenol was the highest, which could explain why the intensity of the smoky aroma in GL was significantly higher than that in the other samples. Furanone is the compound that causes the caramel aroma in coffee. It has a higher OAV in XF and BS, which is consistent with the sensory assessment results, indicating that these samples have a stronger caramel aroma. Nuts, roasted aromas, and woody notes are also key contributors to the aroma in coffee, and are mainly attributed to pyrazines and phenols, such as furfuryl alcohol (OAV ≥ 17), furfural (OAV ≥ 15), and guaiacol (OAV ≥ 8). The coffee also has a faint floral and fruity aroma, which is mainly produced by linalool (OAV ≥ 72) and isovaleric acid (OAV ≥ 72). The OAVs of 4-vinyl-2-methoxyphenol, furanone, linalool, isovaleric acid, 2-ethyl-3, 5-dimethylpyrazine, and 3-hydroxy-2-butanone were all greater than 100, but their contributions to the four kinds of coffee were different, which is one of the most significant reasons for the flavor differences among the four kinds of coffee. Among them, 2-ethyl-3, 5-dimethylpyrazine only had a high OAV in XF. Notably, 4-vinyl-2-methoxyphenol had the highest OAV in all samples and had the greatest impact on the overall flavor of coffee. In contrast, although furfuryl alcohol, γ-butyrolactone, and hydroxyacetone with higher contents had higher concentrations, their OAVs were all no more than 50, which is attributed to their relatively high odor thresholds.

### 3.7. Aroma Recombination

**Figure 6:**
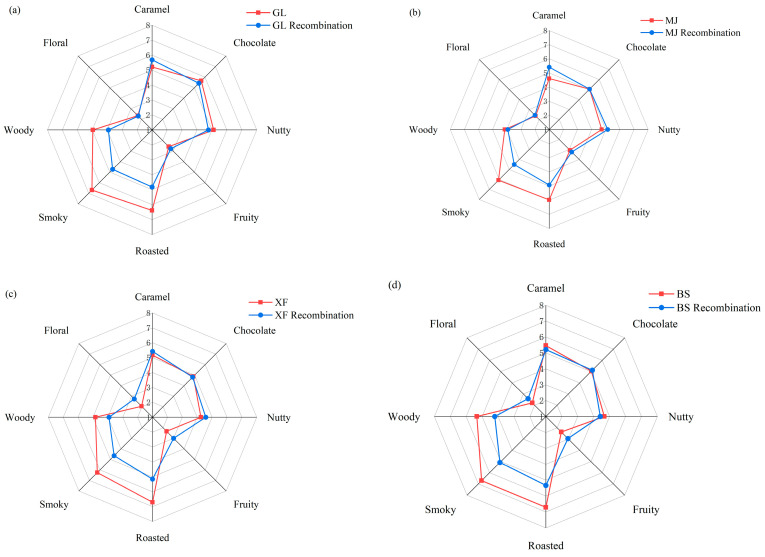
Aroma profiles of four coffee samples, (**a**) GL, (**b**) MJ, (**c**) XF, and (**d**) BS, and their recombination models.

To verify whether the key aroma-active compounds that play a role in the overall aroma of coffee have been correctly identified and quantified, an aroma recombination experiment was conducted. Seventeen odorants with OAVs > 1 were mixed in the artificial odorless matrix. The aroma-reconstituted samples were compared with the original coffee samples through sensory evaluation and eight descriptors representing the perceptible characteristic odors in the coffee aroma. The results are shown in Figure 6. The flavor profiles of the recombinant samples are roughly similar to those of the original samples, indicating that the core compounds of these flavors (such as furans, pyrazines, aldehydes, and ketones, etc.) were accurately identified and quantified. However, the intensities of the smoky, roasted, and woody flavors of the GL, XF, and BS recombinant samples were not as strong as those of the original samples. The reason for this might be that some compounds that provide smoky flavors were not included in the recombination model, or that their concentrations did not reach the sensory threshold. The aroma profile relies on the interaction of multiple trace components, and the addition of only a single compound during recombination leads to a weakened overall perception.

### 3.8. Omission Test

To further determine the contribution of individual compounds to the overall aroma of coffee, a missing model with individual compounds omitted was conducted and evaluated through triangulation tests, as shown in Table 5. γ-butyrolactone, hydroxyacetone, isovaleric acid, 4-vinyl-2-methoxyphenol, furaneol, and guaiacol all showed significance in the four samples. They were identified as key aroma compounds commonly found in coffee, with guaiacol and furaneol demonstrating the highest significance (*p* ≤ 0.001) in BS, consistent with their predominant woody and caramel aroma characteristics in sensory evaluations. In addition, 2,3,5-trimethylpyrazine, 2-ethyl-6-methylpyrazine, and 2-ethyl-3, 5-dimethylpyrazine showed very high significance in GL, MJ, and BS, respectively (*p* ≤ 0.001). The presence of 2,3,5-trimethylpyrazine contributed to the highest chocolate and nutty aroma scores in GL. Linalool showed the greatest significance in MJ, aligning with its highest floral and fruity aroma ratings in sensory evaluations, indicating its substantial contribution to these sensory attributes in MJ. The presence of 2-ethyl-3, 5-dimethylpyrazine is the reason for the high roasted aroma score of XF.

It is worth noting that the OAVs of furfuryl alcohol and furfural are relatively high, but the difference is not significant in the absence model. Furfuryl alcohol and furfural mainly provide the flavors of bread and nuts. We speculate that this substance may work in synergy with other aroma compounds to enhance this odor characteristic. However, when it is absent alone, other compounds can still maintain the overall profile. This may also be because its threshold is relatively low in water, but it may be masked or combined in other media, resulting in a reduction in its actual release.

## 4. Conclusions

This study comprehensively characterized the flavor substances and identified key aroma-active compounds in coffee from four distinct areas with different sensory characteristics. A total of 85 aroma compounds were detected, with furans, ketones, and pyrazines being the predominant volatiles, contributing significantly to the roasted, nutty, and caramel aromas. Notably, furfural, guaiacol, and furaneol exhibited the highest flavor dilution factors (FD = 2187), suggesting their pivotal roles in shaping the overall aroma of the coffee. odor activity value (OAV) analysis further revealed 25 compounds, including 4-vinyl-2-methoxyphenol (OAV = 761 in GL) and furaneol (OAV = 196 in XF and OAV = 250 in BS). Recombination experiments validated the importance of these compounds. Omission experiments confirmed that 2,3,5-trimethylpyrazine is responsible for the chocolate and nutty aromas in GL. Linalool was identified as the causative agent for the prominent floral and fruity notes in MJ, while 2-ethyl-3,5-dimethylpyrazine was demonstrated to be responsible for the dominant roasted aroma in XF. Guaiacol and furaneol were verified as the key odorants contributing to the woody and caramel aroma characteristics in BS. There are significant differences in non-volatile compounds of coffee from different areas, especially in chlorogenic acid and organic acid. GL shows a lower acidity but a higher content of quinic acid, which might be due to degradation caused by roasting. The integrated approach employing GC-O,AEDA, OAV calculation, and recombination/omission tests provides a robust and replicable framework for pinpointing key odorants. Finally, to precisely decouple the effects of origin from processing, future studies should employ identical green coffee beans roasted under instrumentally controlled conditions to specific agtron levels. Such research would further deconvolute the intricate factors shaping coffee aroma and provide more generalizable insights for the industry.

## Figures and Tables

**Table 1:** The L*, a*, and b* values of the CIELAB color space for the coffee samples.

CIELAB Color Space	GL	MJ	XF	BS
L*	25.56 ± 0.21	27.92 ± 0.42	30.98 ± 0.6	28.34 ± 0.05
a*	9.13 ± 0.13	11.86 ± 0.08	12.14 ± 0.07	11.68 ± 0.07
b*	10.6 ± 0.13	14.45 ± 0.17	16.09 ± 0.29	14.74 ± 0.11

**Table 2:** The content of organic acids, caffeine, and chlorogenic acid in coffee.

Compounds	Content (mg/g)			
	GL	MJ	XF	BS
Lactic acid	1.22 ± 0.06 ^ab^	1.18 ± 0.04 ^a^	1.18 ± 0.07 ^a^	1.31 ± 0.04 ^b^
Malic acid	1.24 ± 0.03 ^a^	2.44 ± 0.04 ^b^	2.84 ± 0.03 ^c^	2.83 ± 0.03 ^c^
Citric acid	3.43 ± 0.16 ^a^	4.89 ± 0.07 ^b^	5.94 ± 0.11 ^c^	7.51 ± 0.18 ^d^
Quinic acid	8.34 ± 0.15 ^a^	6.77 ± 0.2 ^b^	6.76 ± 0.13 ^b^	7.51 ± 0.23 ^c^
Succinic acid	ND ^e^	ND	ND	ND
Tartaric acid	ND	ND	ND	ND
Caffeine	12.21 ± 0.13 ^a^	12.05 ± 0.11 ^a^	12.08 ± 0.11 ^a^	11.99 ± 0.17 ^a^
Chlorogenic acid	4.27 ± 0.06 ^a^	21.26 ± 0.45 ^b^	22.33 ± 0.2 ^c^	15.77 ± 0.76 ^d^

Different letters in the same row indicate differences among samples (*p* ≤ 0.05) by Tukey’s test. ^e^ ND, not detected.

**Table 3:** Aroma-active compounds determined by AEDA.

Compounds	Odor Description ^a^	RI		FD ^d^				Identification ^e^
		TG-Wax ^b^	Literature ^c^	GL	MJ	XF	BS	
Pyridine	Smoky	1165	1179	1	3	-	1	MS, RI, Std, O
Limonene	Lemon	1180	1185	1	-	-	-	MS, RI, Std, O
2,4,5-trimethyloxazole	Nuts	1186	1190	1	3	1	-	MS, RI, Std, O
Pyrazine	Roasted potatoes	1193	1210	1	-	-	-	MS, RI, Std, O
Methyl furfuryl ether	Coffee	1224	1243	3	3	9	3	MS, RI, Std, O
3-methyl-3-butene-1-ol	Fruity	1234	1236	3	-	-	-	MS, RI, Std, O
Styrene	Floral	1236	1254	1	-	-	3	MS, RI, Std, O
2-methyltetrahydrofurano-3-one	Nuts	1244	1246	1	-	1	-	MS, RI, Std, O
2-methylpyrazine	Nuts, roasted	1246	1261	1	-	-	1	MS, RI, Std, O
3-hydroxy-2-butanone	Butter	1263	1286	9	-	9	9	MS, RI, Std, O
Hydroxyacetone	Caramel	1278	1275	9	27	9	27	MS, RI, Std, O
2,5-dimethylpyrazine	Roasted potatoes	1300	1303	3	9	9	3	MS, RI, Std, O
2,6-dimethylpyrazine	Nuts, roasted meat	1306	1328	9	9	3	3	MS, RI, Std, O
2-ethylpyrazine	Peanut, woody	1309	1292	1	-	1	1	MS, RI, Std, O
2-hydroxy-3-pentanone	Earthy	1336	1361	1	-	1	1	MS, RI, Std, O
Methylcyclopentenolone	Nuts	1347	1366	3	-	-	-	MS, RI, Std, O
2-ethyl-6-methylpyrazine	Roasted, nuts	1368	1386	3	27	9	27	MS, RI, Std, O
2-ethyl-5-methylpyrazine	Coffee	1374	1383	81	3	3	9	MS, RI, Std, O
2,3,5-trimethylpyrazine	Coffee and cocoa	1389	1405	81	-	27	27	MS, RI, Std, O
2-propyrazine	Vegetables, nuts	1396	-	9	-	-	-	MS, Std, O
Allyl butyrate	Fruity	1414	-	3	-	-	-	MS,O
Acetic acid	Vinegar	1429	1465	3	3	3	1	MS, RI, Std, O
Furfural	Almonds, nuts	1445	1466	2187	2187	2187	2187	MS, RI, Std, O
Acetylacetone peroxide	Nuts	1457	1469	3	-	9	3	MS, RI, Std, O
Furyl methyl sulfide	Onion, spicy	1460	1503	81	-	-	-	MS, RI, Std, O
Tetrahydrofurfuryl alcohol	Caramel	1472	1481	243	-	-	-	MS, RI, Std, O
2-acetylfuran	Cocoa, caramel, Coffee	1483	1479	729	3	729	1	MS, RI, Std, O
Pyrrole	Mold	1487	1505	243	3	-	27	MS, RI, Std, O
Furyl acetate	Sweety	1519	1521	729	3	243	1	MS, RI, Std, O
Linalool	Floral, lemon, rose	1538	1537	3	27	81	9	MS, RI, Std, O
5-methylfurfural	Caramel	1550	1558	243	3	243	3	MS, RI, Std, O
2-acetylpyridine	Barbecue	1570	1590	81	243	-	-	MS, RI, Std, O
Methyl 2-furan propionate	Fruity	1578	1599	3	-	81	-	MS, RI, Std, O
5-methyl-6, 7-dihydro-5H-cyclopentanopyrazine	Nuts, barbecue	1581	1616	1	1	-	9	MS, RI, Std, O
2-(furan-2-ylmethyl)furan	Savory	1584	1628	81	-	-	-	MS, RI, Std, O
γ -butyrolactone	Caramel, roasted nuts	1590	1601	243	81	81	9	MS,RI,O
2-acetylpyrazine	Popcorn	1594	1604	243	81	-	81	MS, RI, Std, O
Butyric acid	Butter, cheese, sour	1610	1628	9	1	1	3	MS, RI, Std, O
2-acetyl-1-methylpyrrole	Soil	1624	1609	1	1	-	-	MS, RI, Std, O
Furfuryl alcohol	Bread	1643	1660	9	9	9	9	MS, RI, Std, O
2(5H)-furanone	Butter	1718	1712	3	-	9	9	MS, RI, Std, O
Methyl salicylate	Mint	1741	1735	1	-	-	-	MS, RI, Std, O
2-hydroxy-2-cyclopentene-1-ketone	Caramel	1747	-	3	27	-	-	MS, Std, O
3,3-dimethacrylic acid	Dairy	1777	1776	3	1	-	1	MS, RI, Std, O
Isovaleric acid	Sour, stinky	1649	1655	243	243	729	243	MS, RI, Std, O
1-(2-furanyl methyl)-1H-pyrrole	Vegetables	1795	1820	729	9	3	3	MS, RI, Std, O
Guaiacol	Woody	1830	1836	2187	2187	2187	2187	MS, RI, Std, O
Ethyl cyclopentenolone	Caramel	1866	1845	9	9	1	1	MS, RI, Std, O
Maltol	Caramel	1935	1943	27	27	9	9	MS, RI, Std, O
2-acetylpyrrole	Barbecue	1941	1949	9	3	3	3	MS, RI, Std, O
Difuryl ether	Coffee	1960	1977	1	9	3	-	MS, RI, Std, O
Phenol	Sweet medicine	1979	1992	729	3	1	-	MS, RI, Std, O
2-pyrrolidine formaldehyde	Mold, coffee	1986	2030	81	1	1	1	MS, RI, Std, O
4-ethyl-2-methoxyphenol	Smokey	1996	2014	729	27	1	27	MS, RI, Std, O
Furaneol	Marshmallows, caramels	2008	2037	2187	2187	2187	2187	MS, RI, Std, O
4-vinyl-2-methoxyphenol	Smoky	2168	2156	243	729	243	2187	MS, RI, Std, O
5-hydroxymethylfurfural	Mold	2483	2509	3	3	1	3	MS, RI, Std, O
Lauryl	Woody	1154	1145	-	1	1	-	MS, RI, Std, O
(+)-limonene	Orange	1178	-	-	1	-	-	MS, Std, O
(E)-3,7-dimethylocta-1,3,6-triene	Sweet herb	1218	1242	-	1	-	-	MS,RI,O
1-hydroxy-2-butanone	Sweet	1351	1375	-	9	3	3	MS, RI, Std, O
2-ethyl-3-methylpyrazine	Nuts	1387	1402	-	1	3	-	MS, RI, Std, O
2-vinylpyrazine	Nuts	1417	1438	-	27	243	9	MS, RI, Std, O
3-ethyl-2, 5-dimethylpirazine	Peanuts	1427	1438	-	27	-	9	MS, RI, Std, O
2-methyl-6-vinyl pyrazine	Roasted	1470	1485	-	3	-	-	MS, RI, Std, O
Furyl formate	Sweet	1480	1504	-	3	-	-	MS,RI,O
Dihydro-2-methyl-3 (2H)-thiophenone	Sulfur	1493	1506	-	3	3	27	MS, RI, Std, O
Benzaldehyde	Almond	1496	1508	-	729	729	729	MS, RI, Std, O
N-methyl-2-pyrrolidine formaldehyde	Roasted nuts	1586	1610	-	3	3	-	MS, RI, Std, O
1-(6-methyl-2-pyrazinyl) acetone	Coffee, cocoa	1671	1676	-	1	3	3	MS,RI,O
Benzyl alcohol	Floral	1847	1877	-	1	3	-	MS, RI, Std, O
Phenylethanol	Rose	1878	1905	-	3	1	-	MS, RI, Std, O
α -ethylene-phenylacetaldehyde	Floral, honey, cocoa	1893	1906	-	1	1	-	MS, RI, Std, O
2-thiophene methanol	Coffee	1911	1890	-	9	27	1	MS, RI, Std, O
3,4-dimethoxystyrene	Fruity, oranges	2019	2014	-	3	-	-	MS, RI, Std, O
Ethyl palmitate	Fruity, creamy	2230	2250	-	3	-	-	MS, RI, Std, O
Indole	Fruity, floral	2403	2412	-	1	-	-	MS, RI, Std, O
2-n-pentylfuran	Fruity	1215	1229	-	-	1	-	MS, RI, Std, O
2,3-dimethylpyrazine	Nuts	1332	1342	-	-	3	3	MS, RI, Std, O
Hexyl formate	Fruity	1340	1382	-	-	1	-	MS, RI, Std, O
2-ethyl-3,5-dimethylpyrazine	Almond	1413	1437	-	-	27	-	MS, RI, Std, O
3-thiol-3-methyl-1-butanol	Roasted vegetables	1631	1658	-	-	3	-	MS, RI, Std, O
3-methylcyclopentane-1,2-dione	Sweet, woody	1800	1781	-	-	3	3	MS, RI, Std, O
2,3-butanediol	Butter	1528	1542	-	-	-	243	MS, RI, Std, O
2, 3-dihydro-3, 5-dihydroxy-6-methyl-4 (H)-pyran-4-one	Smokey	2235	2225	-	-	-	1	MS, RI, Std, O

^a^ Odor description perceived by GC-O analysis. ^b^ Retention indices calculated based on the TG-WAX. ^c^ Retention indices from the literature, https://webbook.nist.gov/chemistry/ (accessed on 12 May 2025). ^d^ Flavor dilution factor: the highest dilution factor obtained by diluting serial times until the odorant was detected via GC-O analysis. ^e^ Identification based on Nist 17 mass spectral database (MS); published retention indices (RI); confirmed by authentic standards (Std); published odor descriptions (O).

**Table 4:** Concentrations, odor thresholds, and odor activity values (OAVs) of aroma compounds in coffee.

Compounds	Standard Curves	Concentration (μg/g) ^a^				Odor Threshold (mg/kg) ^b^	OAV			
		GL	MJ	XF	BS		GL	MJ	XF	BS
Furyl acetate	y = 0.6798x + 0.196	41.53 ± 7.15	-	4.39 ± 0.55	-	- ^c^	-	-	-	-
Furfuryl alcohol	y = 1.7166x + 0.0086	58.95 ± 7.53	49.25 ± 1.76	31.91 ± 2.72	48.03 ± 3.59	1.9	31	26	17	25
Guaiacol	y = 2.1769x + 0.0223	1.14 ± 0.44	0.07 ± 0.03	0.07 ± 0.04	0	0.02	63	12	9	8
2-ethyl-5-methylpyrazine	y = 0.7862x + 0.0037	2.1 ± 0.45	-	-	3.05 ± 0.19	0.04	52	-	-	76
Furyl methyl sulfide	y = 2.1424x − 0.0023	0.44 ± 0.03	-	-	-	-	-	-	-	-
Butyric acid	y = 0.5846x + 0.0008	1.54 ± 0.25	-	-	-	0.204	8	-	-	-
2-acetylpyrazine	y = 0.711x + 0.0018	0.55 ± 0.09	0.59 ± 0.06	-	0.7 ± 0.06	0.06	9	10	-	12
Ethyl cyclopentenolone	y = 0.7003x + 0.0034	2.1 ± 0.94	0.63 ± 0.11	-	-	-	-	-	-	-
Furaneol	y = 0.7x + 0.0003	1.76 ± 0.16	1.75 ± 0.19	1.96 ± 0.45	2.5 ± 0.16	0.01	176	175	196	250
3-hydroxy-2-butanone	y = 0.5683x + 0.0331	5.19 ± 0.16	-	5.94 ± 0.26	7.53 ± 0.62	0.055	94	-	108	137
2, 6-dimethylpyrazine	y = 1.2255x + 0.0071	3.16 ± 0.31	3.01 ± 0.3	-	-	0.4	8	8	-	-
2,3, 5-trimethylpyrazine	y = 1.2279x + 0.0161	1.76 ± 0.38	-	0	1.99 ± 0.09	0.022	77	-	1	90
2-(furan-2-methyl-furan) furan	y = 0.739x + 0.0105	3.08 ± 0.37	-	-	-	-	-	-	-	-
Isovaleric acid	y = 0.8538x + 0.0062	5.01 ± 0.8	13.99 ± 1	10.88 ± 0.1	7.16 ± 0.53	0.07	72	200	155	102
1-(2-furanyl methyl)-1H-pyrrole	y = 2.4452x + 0.0041	0.92 ± 0.23	0.34 ± 0.02	-	-	0.1	9	3	-	-
2-acetylpyrrole	y = 1.6549x + 0.0117	2.76 ± 0.98	-	-	-	58.58	<1	-	-	-
Hydroxyacetone	y = 0.7044x − 0.0126	16.9 ± 0.93	15.12 ± 4.07	31.9 ± 9.1	29.97 ± 8.43	10	2	2	3	3
Furfural	y = 1.2762x + 0.0097	4.32 ± 0.26	15.12 ± 4.08	16.72 ± 3.4	6.26 ± 0.95	0.282	15	62	59	22
2-acetylfuran	y = 1.3788x + 0.0354	3.1 ± 0.4	-	1.65 ± 0.2	-	10	<1	-	<1	-
5-methylfurfural	y = 0.5462x + 0.1844	11.37 ± 2.61	-	23.82 ± 2.13	-	0.5	23	-	48	-
γ -butyrolactone	y = 0.3166x − 0.0365	49.86 ± 4.77	27.45 ± 0.68	16.99 ± 1.14	23.39 ± 1.5	16	3	2	1	1
Maltol	y = 0.8127x − 0.0059	6.1 ± 2.3	2.09 ± 0.29	1.7 ± 0.43	3.49 ± 0.23	0.21	29	10	8	17
Phenol	y = 1.1515x + 0.0375	1.14 ± 0.6	-	-	-	0.5	2	-	-	-
4-vinyl-2-methoxyphenol	y = 1.2822x + 0.0307	2.28 ± 1.17	1.02 ± 0.08	1.59 ± 0.46	1.47 ± 0.1	0.003	761	340	530	491
2-propyrazine	y = 0.9629x − 0.0003	0.28 ± 0.04	-	-	-	0.3	<1	-	-	-
Tetrahydrofurfuryl alcohol	y = 0.7516x − 0.0024	0.83 ± 0.08	-	-	-	-	-	-	-	-
Pyrrole	y = 0.8933x − 0.0063	1.86 ± 0.07	-	-	0.8 ± 0.04	10	<1	-	-	<1
2-acetylpyridine	y = 1.0978x − 0.0047	0.78 ± 0.13	0.58 ± 0.03	-	-	0.019	41	31	-	-
2-pyrrolidine formaldehyde	y = 1.0864x − 0.0021	2.19 ± 0.51	-	-	-	65	<1	-	-	-
4-ethyl-2-methoxyphenol	y = 1.4011x − 0.0003	1.26 ± 0.4	0.19 ± 0.01	-	0.31 ± 0.04	0.016	79	12	-	19
2, 5-dimethylpyrazine	y = 1.3423x + 0.0011	-	3.52 ± 0.35	2.3 ± 0.19	-	0.08	-	44	29	-
2-ethyl-6-methylpyrazine	y = 1.532x − 0.0158	-	2.29 ± 0.05	1.82 ± 0.06	2.88 ± 0.07	0.04	-	57	45	72
2-vinylpyrazine	y = 0.6536 x − 0.0000	-	0.28 ± 0.09	0.29 ± 0.02	0.12 ± 0.01	0.7	-	<1	<1	<1
3-ethyl-2, 5-methylpyrazine	y = 1.3365x − 0.0001	-	0.19 ± 0.09	-	0.04 ± 0.04	0.005	-	38	-	9
Benzaldehyde	y = 0.9385 x − 0.0001	-	0.05 ± 0.01	0.07 ± 0.01	0.04 ± 0.01	0.3	-	<1	<1	<1
Linalool	y = 0.8852x − 0.0047	-	1.33 ± 0.03	1.12 ± 0.12	0.43 ± 0.02	0.006	-	222	187	72
2-hydroxy-2-cyclopentene-1-ketone	y = 0.3923x − 0.0001	-	0.45 ± 0.05	-	-	-	-	-	-	-
2-thiophene methanol	y = 0.657x − 0.0003	-	0.61 ± 0.02	0.5 ± 0.05	-	15	-	<1	<1	-
Difuryl ether	y = 1.1231x − 0.0011	-	0.23 ± 0.04	-	-	-	-	-	-	-
Methyl furfuryl ether	y = 0.8407x − 0.0004	-	-	0.47 ± 0.02	-	-	-	-	-	-
2-ethyl-3, 5-dimethylpyrazine	y = 0.5048x − 0.0001	-	-	0.17 ± 0.01	-	0.001	-	-	170	-
Acetylacetone peroxide	y = 0.5575x + 0.0645	-	-	9.8 ± 0.01	-	-	-	-	-	-
Methyl 2-furan propionate	y = 0.8096x − 0.0004	-	-	0.54 ± 0.01	-	-	-	-	-	-
2(5H)-furanone	y = 0.2708x − 0.0002	-	-	2.76 ± 0.23	3.69 ± 0.27	-	-	-	-	-
Dihydro-2-methyl-3 (2H)-thiophenone	y = 0.8033x − 0.0029	-	-	-	0.9 ± 0.06	-	-	-	-	-
5-methyl-6, 7-dihydro-5H-cyclopentanopyrazine	y = 1.0406x − 0.0014	-	-	-	0.52 ± 0.01	-	-	-	-	-

^a^ Accurate concentration in coffee; means ± SD (n = 3). ^b^ Reference on odor thresholds in water (Van Gemert, 2011 [35]). ^c^ Odor thresholds were unavailable.

**Table 5:** Triangle test results by omission experiments.

No.	Compound Omitted	Significance ^a^			
		GL	MJ	XF	BS
1	Furfuryl alcohol	NS	NS	NS	NS
2	γ -butyrolactone	*	*	*	*
3	Hydroxyacetone	*	*	*	**
4	5-methylfurfural	NS	-	NS	-
5	3-hydroxy-2-butanone	*	-	*	NS
6	Isovaleric acid	**	*	*	*
7	Furfural	NS	NS	NS	*
8	2, 6-dimethylpyrazine	NS	NS	-	-
9	4-vinyl-2-methoxyphenol	**	**	*	*
10	2-ethyl-5-methylpyrazine	**	-	-	**
11	Furaneol	*	*	*	***
12	2,3, 5-trimethylpyrazine	***	-	*	*
13	Butyric acid	NS	-	-	-
14	4-ethyl-2-methoxyphenol	*	*	-	*
15	Guaiacol	*	*	*	***
16	Phenol	NS	-	-	-
17	1-(2-furanyl methyl)-1H-pyrrole	*	*	-	-
18	2-acetylpyridine	*	*	-	-
19	2-acetylpyrazine	NS	NS	-	NS
20	2, 5-dimethylpyrazine	-	*	*	-
21	2-ethyl-6-methylpyrazine	-	***	*	*
22	Maltol	*	*	*	NS
23	Linalool	-	**	*	*
24	3-ethyl-2,5-dimethylpyrazine	-	*	-	*
25	2-ethyl-3, 5-dimethylpyrazine	-	-	***	-

^a^ NS, no significant difference; *, 5% significance level; **, 1% significance level; ***, 0.1% significance level.

## Data Availability

The original contributions presented in this study are included in the article/Supplementary Materials. Further inquiries can be directed to the corresponding author.

## Supplementary Materials

The following supporting information can be downloaded at: https://www.mdpi.com/article/10.3390/foods14183192/s1, Figure S1: The ground coffee powder of four samples; Figure S2: GC-MS chromatograms of the volatile compounds: GL (**a**), MJ (**b**), XF (**c**), BS (**d**); Figure S3: LC-MS chromatograms of the non-volatile compounds from four coffee: lactic acid (**a**), malic acid (**b**), Citric acid (**c**), quinic acid (**d**), Succinic acid (**e**), tartaric acid (**f**), Caffeine (**g**), Chlorogenic acid (**h**). Table S1: Standard curves of non-volatile compounds detected in four samples.

## Abbreviations

The following abbreviations are used in this manuscript:

SAFE	Solvent-assisted flavor evaporation
GC-MS	Gas chromatography–mass spectrometry
GC-O	Gas chromatography–olfactometry
AEDA	Aroma extract dilution analysis
OAV	Odor activity value
LC-MS	Liquid chromatography–mass spectrometry
HPLC	High-performance liquid chromatography
QDA	Quantitative descriptive analysis

## References

[1] Martins V.d.C., Godoy R.L.d.O., Gouvêa A.C.M.S., Santiago M.C.P.d.A., Borguini R.G., Braga E.C.d.O., Pacheco S., Nascimento L.d.S.d.M.D. (2018). Fraud investigation in commercial coffee by chromatography. Food Qual. Saf..

[2] Ongo E.A., Montevecchi G., Antonelli A., Sberveglieri V., Iii F.S. (2020). Metabolomics fingerprint of Philippine coffee by SPME-GC-MS for geographical and varietal classification. Food Res. Int..

[3] Knysak D. (2017). Volatile compounds profiles in unroasted Coffea arabica and Coffea canephora beans from different countries. Food Sci. Technol..

[4] Cui D.D., Liu Y., Chen Y.P., Feng X., Lu Y., Yu B. (2020). Application of SPME-GC-TOFMS, E-nose, and sensory evaluation to investigate the flavor characteristics of Chinese Yunnan coffee at three different conditions (beans, ground powder, and brewed coffee). Flavour Fragr. J..

[5] Toledo P.R., Pezza L., Pezza H.R., Toci A.T. (2016). Relationship Between the Different Aspects Related to Coffee Quality and Their Volatile Compounds. Compr. Rev. Food Sci. Food Saf..

[6] Amalia F., Aditiawati P., Yusianto, Putri S.P., Fukusaki E. (2021). Gas chromatography/mass spectrometry-based metabolite profiling of coffee beans obtained from different altitudes and origins with various postharvest processing. Metabolomics.

[7] Bichlmaier C., Fröhlich S.M., Brychcy V., Graßl A., Behrens M., Lang R. (2024). Contribution of mozambioside roasting products to coffee’s bitter taste. Food Chem..

[8] Rune C.J., Giacalone D., Steen I., Duelund L., Münchow M., Clausen M.P. (2023). Acids in brewed coffees: Chemical composition and sensory threshold. Curr. Res. Food Sci..

[9] Yeager S.E., Batali M.E., Guinard J.-X., Ristenpart W.D. (2023). Acids in coffee: A review of sensory measurements and meta-analysis of chemical composition. Crit. Rev. Food Sci. Nutr..

[10] Sunarharum W.B., Williams D.J., Smyth H.E. (2014). Complexity of coffee flavor: A compositional and sensory perspective. Food Res. Int..

[11] Shi X., Li Y., Huang D., Chen S., Zhu S. (2025). Characterization and discrimination of volatile compounds in roasted Arabica coffee beans from different origins by combining GC-TOFMS, GC-IMS, and GC-E-Nose. Food Chem..

[12] Portela C.d.S., de Almeida I.F., Mori A.L.B., Yamashita F., Benassi M.d.T. (2021). Brewing conditions impact on the composition and characteristics of cold brew Arabica and Robusta coffee beverages. LWT.

[13] Marek G., Dobrzański B., Oniszczuk T., Combrzyński M., Ćwikła D., Rusinek R. (2020). Detection and differentiation of volatile compound profiles in roasted coffee arabica beans from different countries using an electronic nose and GC-MS. Sensors.

[14] Jeong H., Yoon S., Jo S.M., Hong S.J., Kim Y.J., Kim J.K., Shin E. (2023). Chemical sensory investigation in green and roasted beans *Coffea arabica* L. (cv. *Yellow bourbon*) by various brewing methods using electronic sensors. J. Food Sci..

[15] Pua A., Lau H., Liu S.Q., Tan L.P., Goh R.M.V., Lassabliere B., Leong K.-C., Sun J., Cornuz M., Yu B. (2020). Improved detection of key odourants in Arabica coffee using gas chromatography-olfactometry in combination with low energy electron ionisation gas chromatography-quadrupole time-of-flight mass spectrometry. Food Chem..

[16] Dong W., Tan L., Zhao J., Hu R., Lu M. (2015). Characterization of fatty acid, amino acid and volatile compound compositions and bioactive components of seven coffee (*Coffea robusta*) cultivars grown in Hainan Province, China. Molecules.

[17] Freitas A.M.C., Mosca A.I. (1999). Coffee geographic origin—An aid to coffee differentiation. Food Res. Int..

[18] Nie R., Zhang C., Liu H., Wei X., Gao R., Shi H., Zhang D., Wang Z. (2024). Characterization of key aroma compounds in roasted chicken using SPME, SAFE, GC-O, GC–MS, AEDA, OAV, recombination-omission tests, and sensory evaluation. Food Chem. X.

[19] Xie X., Wang Y., Wen B., Tian J., Cheng Z., Tang S., Nie Y., Wu X., Guo X., Li B. (2025). Characterization and metabolism pathway of volatile compounds in blueberries of different varieties and origins analyzed via HS-GC-IMS and HS-SPME-GC–MS. Food Chem..

[20] Lau H., Liu S.Q., Xu Y.Q., Lassabliere B., Sun J., Yu B. (2018). Characterising volatiles in tea (*Camellia sinensis*). Part I: Comparison of headspace-solid phase microextraction and solvent assisted flavour evaporation. LWT.

[21] Zhai H., Dong W., Tang Y., Hu R., Yu X., Chen X. (2024). Characterization of the volatile flavour compounds in Yunnan Arabica coffee prepared by different primary processing methods using HS-SPME/GC-MS and HS-GC-IMS. LWT.

[22] Yu M., Li T., Song H. (2022). Characterization of key aroma-active compounds in four commercial oyster sauce by SGC/GC × GC–O–MS, AEDA, and OAV. J. Food Compos. Anal..

[23] Pang X., Yin H., Li J., Shi Y., Yang Z. (2025). Molecular insights into the contribution of oak barrel aging to the aroma of beer with high alcohol content using SAFE-GC-O/AEDA and OAV calculation. Food Chem..

[24] Czerny M., Mayer F., Grosch W. (1999). Sensory study on the character impact odorants of roasted arabica coffee. J. Agric. Food Chem..

[25] Moon S.A., Wongsakul S., Kitazawa H., Saengrayap R. (2025). Impact of Roasting and Storage Conditions on the Shelf Stability of Thai Arabica Coffee. J. Agric. Food Res..

[26] Tsai C.F., Jioe I.P.J. (2021). The analysis of chlorogenic acid and caffeine content and its correlation with coffee bean color under different roasting degree and sources of coffee (*Coffea arabica typica*). Processes.

[27] Alcantara G.M., Martins L.C., Gomes W.P., Dresch D., Rocha F.R., Melchert W.R. (2025). Effect of roasting on chemical composition of coffee. Food Chem..

[28] Santanatoglia A., Alessandroni L., Fioretti L., Sagratini G., Vittori S., Maggi F., Caprioli G. (2023). Discrimination of Filter Coffee Extraction Methods of a Medium Roasted Specialty Coffee Based on Volatile Profiles and Sensorial Traits. Foods.

[29] Kulapichitr F., Borompichaichartkul C., Suppavorasatit I., Cadwallader K.R. (2019). Impact of drying process on chemical composition and key aroma components of Arabica coffee. Food Chem..

[30] Angeloni S., Mustafa A.M., Abouelenein D., Alessandroni L., Acquaticci L., Nzekoue F.K., Petrelli R., Sagratini G., Vittori S., Torregiani E. (2021). Characterization of the Aroma Profile and Main Key Odorants of Espresso Coffee. Molecules.

[31] Seninde D.R., Chambers E. (2020). Coffee flavor: A review. Beverages.

[32] Wei F., Furihata K., Hu F., Miyakawa T., Tanokura M. (2011). Two-dimensional 1H–13C nuclear magnetic resonance (NMR)-based comprehensive analysis of roasted coffee bean extract. J. Agric. Food Chem..

[33] Santanatoglia A., Angeloni S., Caprioli G., Fioretti L., Ricciutelli M., Vittori S., Alessandroni L. (2024). Comprehensive investigation of coffee acidity on eight different brewing methods through chemical analyses, sensory evaluation and statistical elaboration. Food Chem..

[34] Song H., Liu J. (2018). GC-O-MS technique and its applications in food flavor analysis. Food Res. Int..

[35] Van Gemert L.J. (2011). Compilations of Odour Threshold Values in Air, Water and Other Media.

[36] Wang M.-Q., Ma W.-J., Shi J., Zhu Y., Lin Z., Lv H.-P. (2020). Characterization of the key aroma compounds in Longjing tea using stir bar sorptive extraction (SBSE) combined with gas chromatography-mass spectrometry (GC–MS), gas chromatography-olfactometry (GC-O), odor activity value (OAV), and aroma recombination. Food Res. Int..

