# Supplementary material for: Identification of Key Aroma-Active Compounds in Commercial Coffee Using GC-O/AEDA and OAV Analysis
Source: Foods. 2025 Sep 13;14(18):3192. doi: 10.3390/foods14183192 (PMC12469716; doi:10.3390/foods14183192)
Supplement: Supplementary file 1 [file foods-14-03192-s001.zip › foods-3811327-supplementary.pdf]

Supplementary Figure S1. The ground coffee powder of four samples.

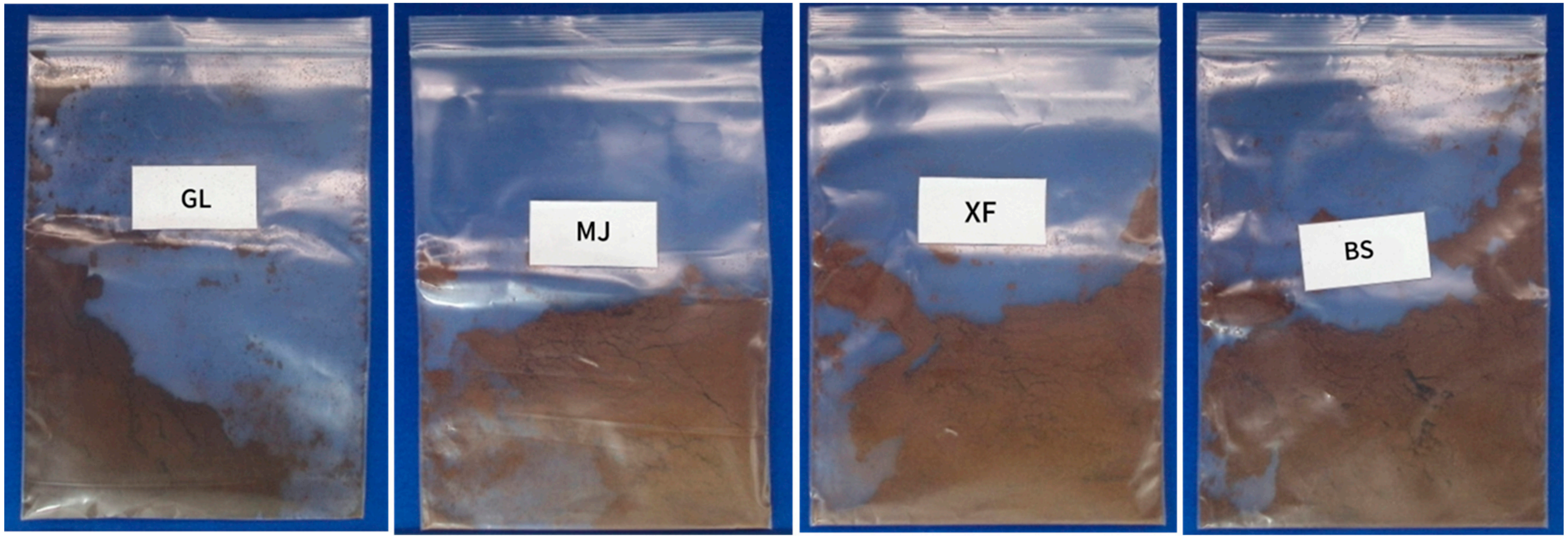

Supplementary Figure S2. GC-MS chromatograms of the volatile compounds: GL(a)、MJ(b)、XF(c)、BS(d).

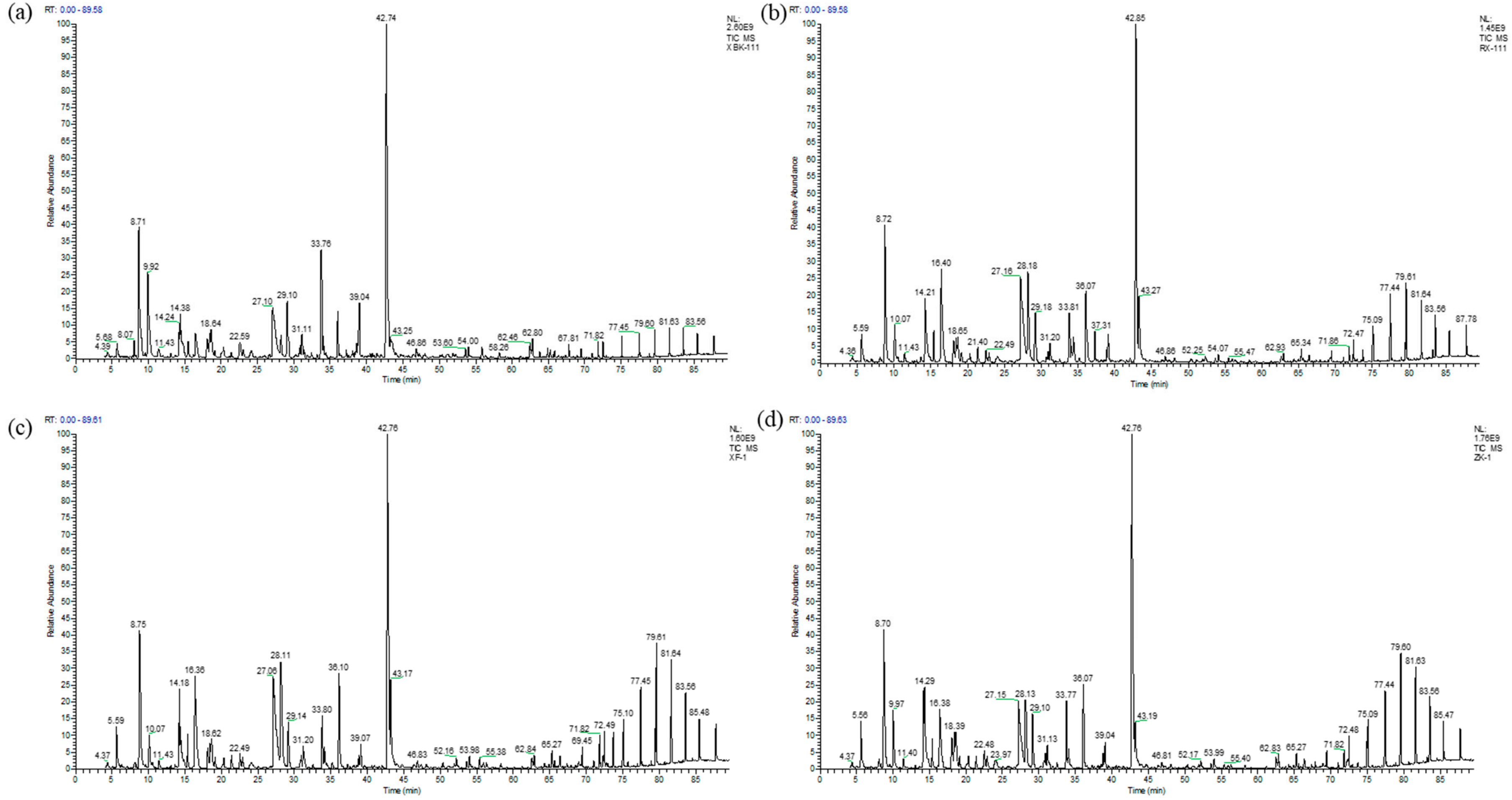

Supplementary Figure S3. LC-MS chromatograms of the non-volatile compounds from four coffee: lactic acid(a)、malic acid(b)、Citric acid(c)、quinic acid(d)、Succinic acid(e)、tartaric acid(f)、Caffeine(g)、Chlorogenic acid(h).

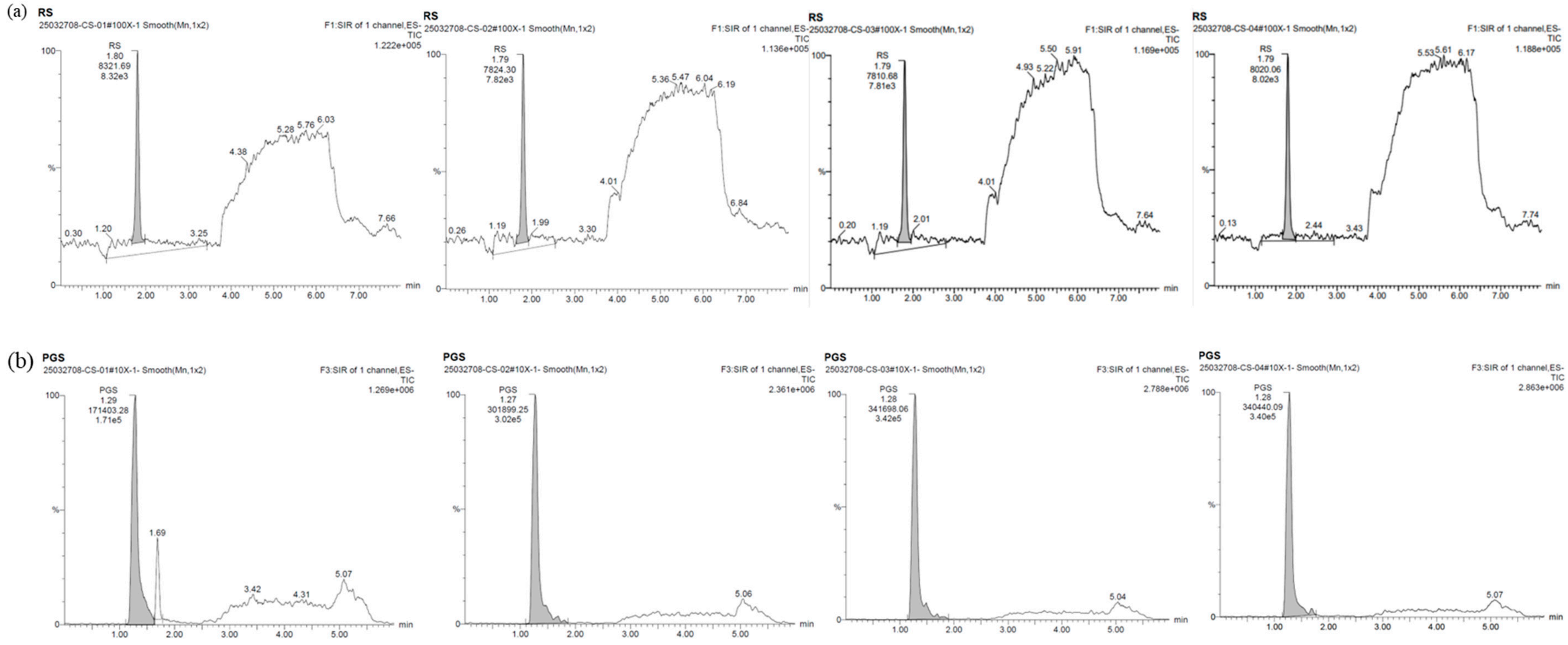

(c)

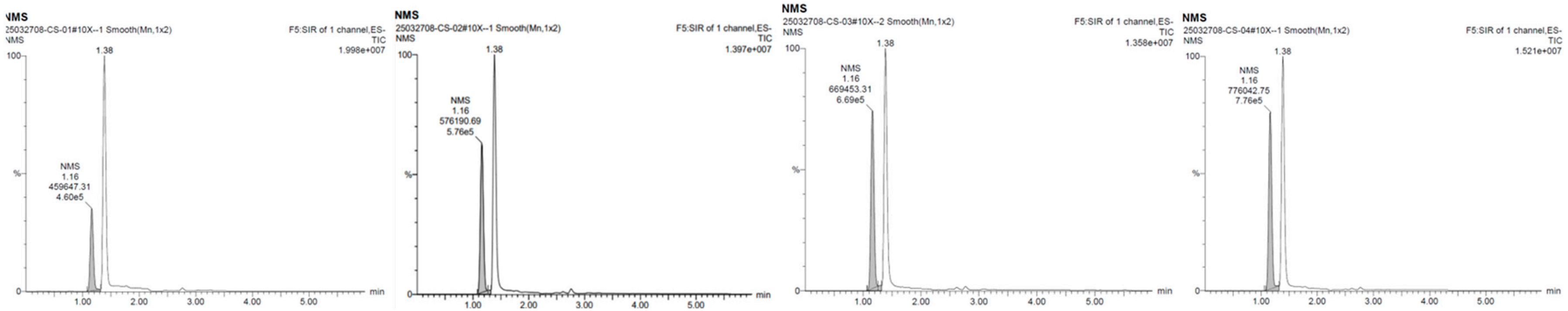
NMS
25032708-CS-01#10X--1 Smooth(Mn,1x2)
NMS
F5:SIR of 1 channel,ES-
TIC
1.998e+007
1.38
NMS
1.16
459647.31
4.60e5
25032708-CS-02#10X--1 Smooth(Mn,1x2)
F5:SIR of 1 channel,ES-
TIC
1.397e+007
NMS
1.16
576190.69
5.76e5
25032708-CS-03#10X--2 Smooth(Mn,1x2)
F5:SIR of 1 channel,ES-
TIC
1.358e+007
NMS
1.16
669453.31
6.69e5
25032708-CS-04#10X--1 Smooth(Mn,1x2)
F5:SIR of 1 channel,ES-
TIC
1.521e+007
NMS
1.16
776042.75
7.76e5
100
%
0
1.00
2.00
3.00
4.00
5.00
min

(d)

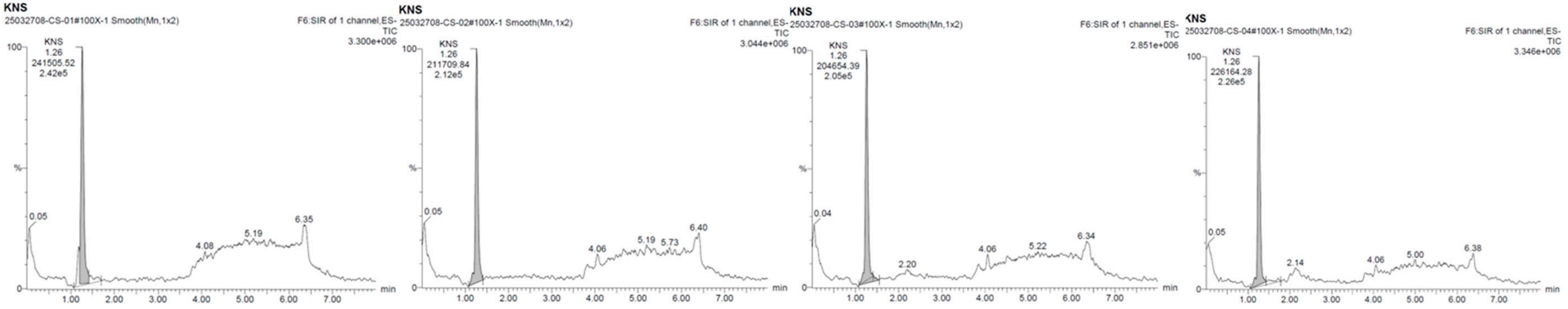
KNS
25032708-CS-01#100X-1 Smooth(Mn,1x2)
F6:SIR of 1 channel,ES-
TIC
3.300e+006
KNS
1.26
241505.52
2.42e5
0.05
4.08
5.19
6.35
25032708-CS-02#100X-1 Smooth(Mn,1x2)
F6:SIR of 1 channel,ES-
TIC
3.044e+006
KNS
1.26
211709.84
2.12e5
0.05
4.06
5.19
5.73
6.40
25032708-CS-03#100X-1 Smooth(Mn,1x2)
F6:SIR of 1 channel,ES-
TIC
2.851e+006
KNS
1.26
204654.39
2.05e5
0.04
2.20
4.06
5.22
6.34
25032708-CS-04#100X-1 Smooth(Mn,1x2)
F6:SIR of 1 channel,ES-
TIC
3.346e+006
KNS
1.26
226164.28
2.26e5
0.05
2.14
4.06
5.00
6.38
100
%
0
1.00
2.00
3.00
4.00
5.00
6.00
7.00
min

(e)

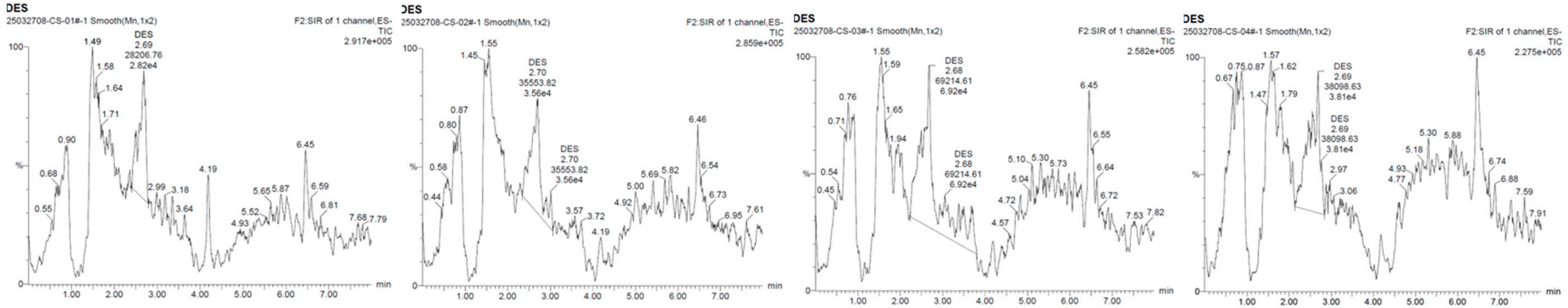
DES
25032708-CS-01#-1 Smooth(Mn,1x2)
F2:SIR of 1 channel,ES-
TIC
2.917e+005
DES
2.69
28206.76
2.82e4
DES
25032708-CS-02#-1 Smooth(Mn,1x2)
F2:SIR of 1 channel,ES-
TIC
2.859e+005
DES
2.70
35553.82
3.56e4
DES
25032708-CS-03#-1 Smooth(Mn,1x2)
F2:SIR of 1 channel,ES-
TIC
2.582e+005
DES
2.68
69214.61
6.92e4
DES
25032708-CS-04#-1 Smooth(Mn,1x2)
F2:SIR of 1 channel,ES-
TIC
2.275e+005
DES
2.69
38098.63
3.81e4
min

(f)

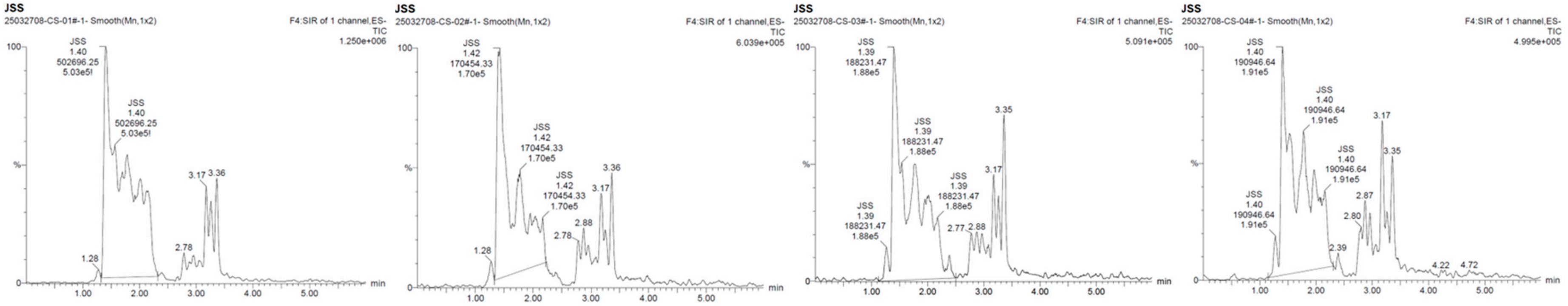
JSS
25032708-CS-01#-1- Smooth(Mn,1x2)
F4:SIR of 1 channel,ES-
TIC
1.250e+006
JSS
1.40
502696.25
5.03e5!
JSS
25032708-CS-02#-1- Smooth(Mn,1x2)
F4:SIR of 1 channel,ES-
TIC
6.039e+005
JSS
1.42
170454.33
1.70e5
JSS
25032708-CS-03#-1- Smooth(Mn,1x2)
F4:SIR of 1 channel,ES-
TIC
5.091e+005
JSS
1.39
188231.47
1.88e5
JSS
25032708-CS-04#-1- Smooth(Mn,1x2)
F4:SIR of 1 channel,ES-
TIC
4.995e+005
JSS
1.40
190946.64
1.91e5
min

(g)

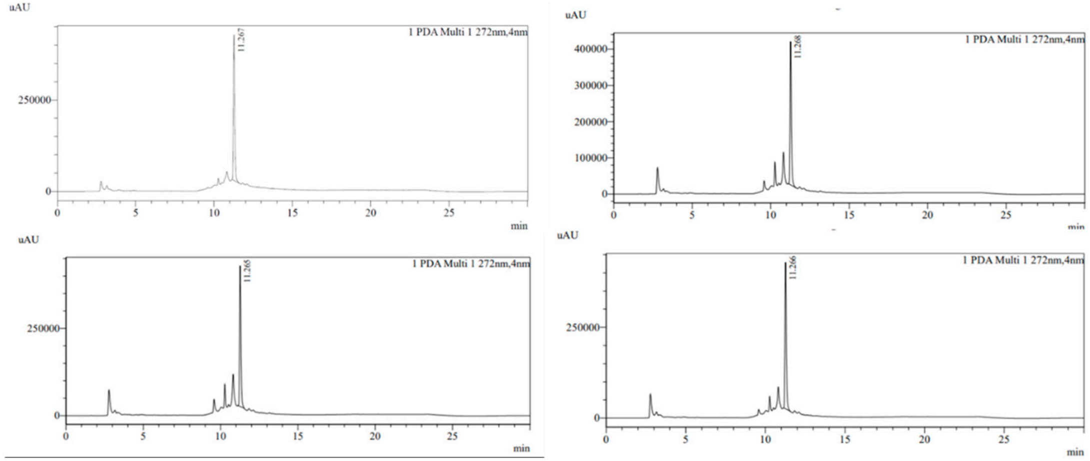
uAU
1 PDA Multi 1 272nm,4nm
11.267
11.268
11.265
11.266
min

(h)

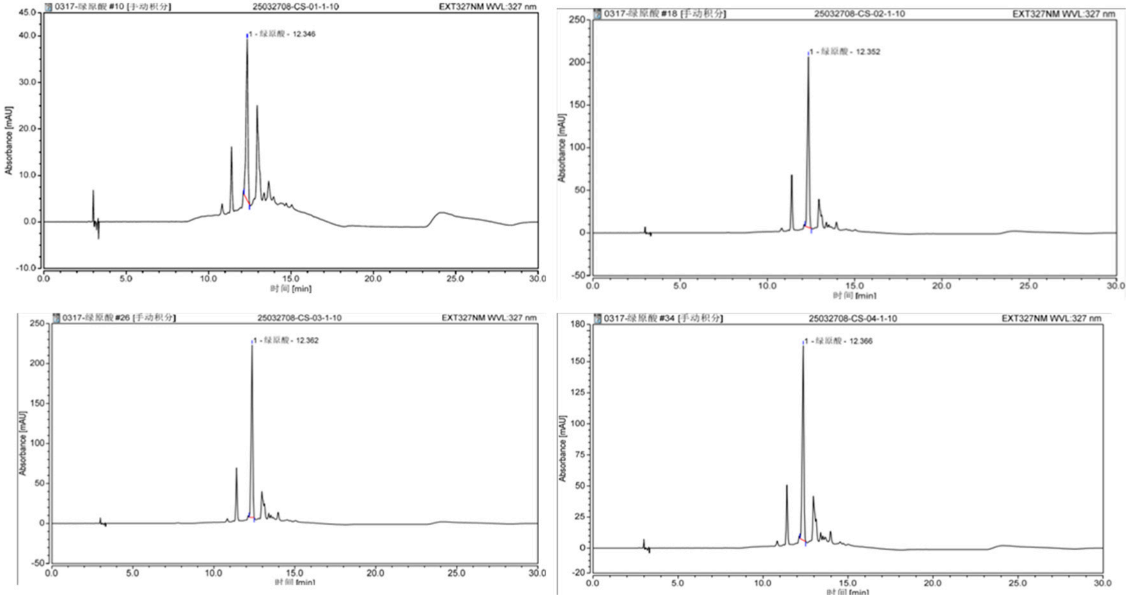
0317-绿原酸 #10 [手动积分]
25032708-CS-01-1-10
EXT327NM WVL:327 nm
1 - 绿原酸 - 12.346
0317-绿原酸 #18 [手动积分]
25032708-CS-02-1-10
1 - 绿原酸 - 12.352
0317-绿原酸 #26 [手动积分]
25032708-CS-03-1-10
1 - 绿原酸 - 12.362
0317-绿原酸 #34 [手动积分]
25032708-CS-04-1-10
1 - 绿原酸 - 12.366
Absorbance [mAU]
时间 [min]

Supplementary Table S1

Standard curves of non-volatile compounds detected in four samples.

No.	Compounds	Standard curves	R^2
1	lactic acid	$y = -2043.43x^2 + 49403.3x + 1719.12$	$R^2 = 0.9999$
2	malic acid	$y = -3436.03x^2 + 125538x + 12282.8$	$R^2 = 0.9999$
3	Citric acid	$y = -3479.6x^2 + 124358x + 44356.4$	$R^2 = 0.9991$
4	quinic acid	$y = -39185.7x^2 + 277625x + 32212.2$	$R^2 = 0.9997$
5	Succinic acid	$y = -1549.34x^2 + 135597x + 7993.05$	$R^2 = 0.9992$
6	tartaric acid	$y = 405.981x^2 + 100767x + -8418.36$	$R^2 = 0.9998$
7	Caffeine	$y = 0.0591x + 0.5523$	$R^2 = 0.9999$
8	Chlorogenic acid	$y = 31845.0x + 5682.45$	$R^2 = 0.9999$
